# Age-specific reference values for unstimulated whole saliva flow rate in primary Sjögren’s syndrome: a cross-sectional study

**DOI:** 10.1186/s12903-026-09160-2

**Published:** 2026-07-08

**Authors:** Yang Yang, Xin-Xiang Huang, Dan-Li Meng, Yi-Jia Huang, Yan-Ting Yang, Rong-Xiu Huo, Jin-Ying Lin

**Affiliations:** Department of Rheumatology and Immunology, People’s Hospital of Guangxi Zhuang Autonomous Region, Nanning, 530016 China

**Keywords:** Sjögren’s syndrome, Sjögren’s disease, Saliva, Hyposalivation, Age of onset, Reference value

## Abstract

**Background:**

The clinical phenotype of primary Sjögren’s syndrome (pSS) varies with age at onset, yet evidence on age and unstimulated whole saliva (UWS) flow rate in pSS is limited and conflicting. This study describes the age-specific distribution of UWS flow rate in pSS patients and provides reference values for clinical use.

**Methods:**

This retrospective cross-sectional study included pSS patients diagnosed per 2016 ACR/EULAR criteria, stratified by age at diagnosis: early-onset (≤ 35 years), middle-onset (35–65 years), and elderly-onset (≥ 65 years). UWS flow rate was compared across groups. Linear regression assessed the age-UWS association, adjusting for sex, pre-diagnostic period, and anticholinergic use. For each age group, we calculated the 5th, 25th, 50th, 75th, and 95th percentiles of UWS flow rate as reference values.

**Results:**

Of 338 pSS patients (early-onset ≤ 35 years: 40; middle-onset 35–65 years: 193; elderly-onset ≥ 65 years: 105), UWS flow rate decreased significantly with age. Each 10-year increase was associated with a 0.04 mL/min decrease (β= -0.04, 95% CI: -0.06 to -0.03, *P* < 0.001), independent of sex, pre-diagnostic period, and anticholinergic use. The median UWS flow rate was 0.29[0.20–0.47] mL/min in the early-onset group, 0.20[0.10–0.39] mL/min in the middle-onset group, and 0.14[0.05–0.22] mL/min in the elderly-onset group, with significant differences between early- and middle-onset (*P* = 0.013) and between middle- and elderly-onset (*P* < 0.001). Age-specific percentiles were established as reference values.

**Conclusion:**

UWS flow rate in pSS patients declines with age, independent of confounders. The age-specific reference values provided herein facilitate longitudinal monitoring and interpretation of UWS measurements in clinical practice.

**Supplementary Information:**

The online version contains supplementary material available at https://doi.org/10.1186/s12903-026-09160-2.

## Key Points

• UWS flow rate in pSS patients decreases significantly with age (β= -0.04 per 10 years, *P* < 0.001), independent of confounders.

• Median UWS flow rate showed a stepwise decline across age groups: 0.29 mL/min (early-onset), 0.20 mL/min (middle-onset), and 0.14 mL/min (elderly-onset), with significant differences between adjacent groups (*P* = 0.013 and *P* < 0.001).

• This study provides the first age-specific percentile-based reference values for UWS flow rate, enabling individualized patient monitoring.

**Supplementary Information:**

The online version contains supplementary material available at https://doi.org/10.1186/s12903-026-09160-2.

## Background

Primary Sjögren’s syndrome (pSS)-now also designated primary Sjögren’s disease according to the 2023 International Rome Consensus [1]-is a connective tissue disease characterized by a wide range of clinical manifestations, from exocrine gland to systemic extra-glandular involvement. The mean age at diagnosis was reported as 52.2 years in a 2021 study [2].

Clinically, the disease most commonly manifests as exocrine gland dysfunction, resulting in the hallmark symptoms of xerostomia (subjective dry mouth) and ocular dryness [3]. These sicca symptoms constitute the primary clinical clue that prompts suspicion for pSS. In addition, the clinical phenotype of pSS varies with age of onset. Older age at onset (≥ 65 years old) is associated with a lower frequency of arthritis and lower prevalence of positive salivary glands ultrasound findings [4]. In contrast, early-onset pSS (≤ 35 years old) presents a distinct profile, with more severe symptoms, heightened activation of circulating T and B cells, and a poorer prognosis [5]. The occurrence of subjective dry mouth was less frequent in early-onset pSS when compared to both middle-aged pSS controls (*P* < 0.05) [6, 7] and elderly-onset pSS patients (*P* < 0.05) [7]. However, two other studies have indicated that there was no significant difference in the incidence of subjective dry mouth between patients with early- and late-onset pSS [5, 8]. Given the extremely low specificity (9%) of subjective xerostomia in detecting hyposalivation [9], objective assessment of salivary function is essential. Unstimulated whole saliva (UWS) flow rate offers a simple, non-invasive method for this purpose.

Estimates of UWS flow rate in the general adult population vary across studies. Bergdahl [10]reported mean UWS flow rate of 0.33 ± 0.26 mL/min in men and 0.26 ± 0.21 mL/min in women in a population-based study of 1427 adults aged 20–69 years. Fenoll-Palomares et al. [11] found a median UWS flow rate of 0.48 mL/min (range: 0.1-2.0 mL/min) in 159 healthy volunteers. Jehl-Pietri et al. [12] documented mean UWS flow rate of 0.42 mL/min in males and 0.37 mL/min in females, proposing 0.1 mL/min as the cut-off for hyposalivation diagnosis. These data highlight considerable inter-individual variation in salivary flow, with reported means ranging from 0.26 to 0.48 mL/min across different populations [10–13].

According to the latest 2016 classification criteria for pSS-in which subjective symptom of dry mouth is no longer part of the scoring items-the UWS flow rate is included as one of the objective measures, with a value of ≤ 0.1 mL/min contributing 1 point to the total score [14]. Age-related variations in salivary flow have been observed in the general population [15]. Whether this age-related pattern also exists in pSS remains unclear, as evidence on the effect of age on UWS flow rate in pSS is limited and conflicting. One report found no significant differences in UWS flow rate across age groups [4], whereas a recent large cohort analysis confirmed an age-related decline in UWS flow rate in SS patients [16]. Meanwhile, two other investigations have reached divergent conclusions regarding the impact of age on UWS threshold: one suggested that diagnostic performance varied with age [9], while the other found no evidence that age affected the optimal threshold [17]. Although age has been suggested to play a role in pSS, few studies have systematically investigated the age-specific distribution of UWS flow rate in this population or provided age-stratified reference values for clinical use. Such data would be particularly valuable given the distinct clinical profiles of early- and elderly-onset pSS and the potential need for age-appropriate interpretation of UWS measurements.

Therefore, the aims of this study are to describe the distribution of UWS flow rate across different ages in a cohort of patients with pSS, and to provide age-specific reference values that may assist clinicians in interpreting salivary function in clinical practice.

## Methods

### Study population

This is a retrospective study. Patients with suspected SS (presenting with symptoms such as dry mouth, dry eyes, or various extra-glandular symptoms) who were treated at the People’s Hospital of Guangxi Zhuang Autonomous Region from 2021 to May 2025 were included. According to the 2016 ACR/EULAR classification criteria [14], pSS diagnosis requires a total score ≥ 4 points, based on: anti-SSA positivity (3 points); focal lymphocytic sialadenitis with focus score (FS) ≥ 1/4 mm² on minor salivary gland biopsy (MSGB) (3 points); Schirmer’s test ≤ 5 mm/5min (1 point); UWS flow rate ≤ 0.1 mL/min (1 point); and ocular staining score (OSS) ≥ 5 or van Bijsterveld score ≥ 4 in at least one eye (1 point). OSS is not performed in our institution. However, in the 2016 ACR/EULAR classification criteria, OSS is a minor variable (1 point); therefore, even if data on OSS were missing, a total score of ≥ 4 points was still sufficient to confirm the diagnosis of pSS. Exclusions included patients with associated SS, individuals who met the exclusion criteria outlined in the 2016 ACR/EULAR classification criteria, patients with an unclear diagnosis, those with incomplete medical records, and those still being evaluated for pSS. As an example, if patients had a score of 3 based on the ACR/EULAR criteria, missing OSS or FS data could alter their pSS diagnosis. In these situations, we excluded such patients from our study due to diagnostic uncertainty.

Patients were divided into three groups according to age at the time of pSS diagnosis: elderly-onset group (≥ 65 years), middle-onset group (> 35 and < 65 years), and early-onset group (≤ 35 years).

This study was approved by the Ethics Committee of the People’s Hospital of Guangxi Zhuang Autonomous Region (ethics approval number: KY-IIT-2025-146). All patients provided informed consent prior to participation.

### Data collection

We collected the following data using a standardized method during diagnosis: age, gender, pre-diagnostic period (defined as the time from symptom onset to diagnosis), presence of hypertension, diabetes mellitus, and depression/anxiety, use of anticholinergic medications, UWS flow rate, Schirmer’s results, MSGB findings, and test results for antinuclear antibodies (ANA), anti-SSA antibodies, and anti-SSB antibodies.

As recommended [18], all participants underwent UWS flow rate evaluation in a quiet and relaxed environment. The collection lasted 10 min between 9:30 am and 11:30 am, after a 3‑hour fast, at least 30 min without water intake or oral hygiene, and no smoking or coffee since the previous day. Patients swallowed residual saliva before the test, then allowed saliva to flow passively from the lower lip into a container without chewing, swallowing, or orofacial movements. Saliva volume was measured using a 0.1 mL‑calibrated syringe.

### Statistical analysis

SPSS 22.0 was used for data analysis. Continuous variables with a normal distribution were expressed as mean (standard deviation, SD), and comparisons between groups were performed using analysis of variance. Non-normally distributed data were presented as median (P25, P75), and inter-group comparisons were performed via the Mann-Whitney U test. The Kolmogorov-Smirnov test was employed to determine the normality of distribution. Categorical variables were expressed as case number and percentage, and inter-group comparisons were made using the Chi-squared test or Fisher’s exact test.

A linear regression model was applied to evaluate the association between age and UWS flow rate. In this model, UWS flow rate served as the dependent variable and age as the independent variable. The regression coefficient (β) with its 95% CI was calculated to quantify the change in UWS flow rate per 10-year increase in age. Multivariable linear regression was additionally performed to adjust for potential confounders, including sex, pre-diagnostic period, and use of anticholinergic medications.

To describe the distribution of UWS flow rate across different age groups, percentiles (5th, 25th, 50th, 75th, and 95th) were calculated for each predefined age category. *P* value < 0.05 was considered statistically significant.

## Results

### Characterization of the three groups

The general characteristics of the three groups are summarized in Table 1. A total of 338 consecutive patients with pSS were included, 40 early-onset (11.8%), 193 middle-onset (57.1%), and 105 elderly-onset (31.1%). The majority of patients were female, with no significant differences between groups. The median pre-diagnostic period for the overall cohort was 6 [1–36] months. Pre-diagnostic period was shorter in early-onset (3.5 months) than middle-onset (6.0 months, *P* = 0.03).

**Table 1:** Characteristics of the three groups. The horizontal line that separates the column headers from the first row of data appears in the wrong position, causing the “Female” row to be misaligned

Female	Early-onset group (*n* = 40)	Middle-onset group (*n* = 193)	Elderly-onset group (*n* = 105)	*P*-value ^a^	*P*-value ^b^
	36/40 (90.0%)	171/193 (88.6%)	88/105 (83.8%)	1.00	0.24
Age at pSS diagnosis, years	28[23–33]	55[50–59]	71[66–74]	< 0.001	< 0.001
Pre-diagnostic period, months	3.5[1.0-11.5]	6.0[1.0–36.0]	7.0[1.0–24.0]	0.03	0.85
Hypertension	0/40 (0%)	20/193 (10.4%)	29/105 (27.6%)	0.03	< 0.001
Diabetes mellitus	0/40 (0%)	7/193 (3.6%)	12/105 (11.4%)	0.60	0.008
Depression/anxiety	2/40 (5.0%)	7/193 (3.6%)	2/105 (1.9%)	0.65	0.50
Anticholinergic medication use	0/40 (0%)	9/193 (4.7%)	2/105 (1.9%)	0.36	0.33
Schirmer (mm/5min)	3.0[0–15.0]	2.0[0–5.0]	2.0[0–5.0]	0.35	0.99
Schirmer ≤5 mm/5min	25/39 (64.1%)	160/187 (85.6%)	87/99 (87.9%)	0.006	0.48
UWS flow (mL/min)	0.29[0.20–0.47]	0.20[0.10–0.39]	0.14[0.05–0.22]	0.013	< 0.001
UWS flow rate ≤ 0.1mL/min	6/40 (15.0%)	60/193 (31.1%)	68/105 (64.8%)	0.04	0.012
Positive Anti-ANA	22/40 (55.0%)	104/193 (53.9%)	73/105 (69.5%)	0.89	0.009
Positive Anti-SSA	30/40 (75.0%)	129/193 (66.8%)	79/105 (75.2%)	0.31	0.13
Positive Anti-SSB	12/40 (30.0%)	35/193 (18.1%)	30/105 (28.6%)	0.08	0.037
Focus score ≥ 1 on MSGB	26/27 (96.2%)	99/103 (96.1%)	47/51 (92.2%)	0.26	0.43

*pSS* primary Sjögren’s syndrome, Early-onset ≤ 35 years; middle-onset: >35 and < 65 years; Elderly-onset ≥ 65 years; *ANA* anti-nuclear antibodies, *MSGB* minor salivary gland biopsy, *UWS* unstimulated whole saliva; *P*^a^: early-onset group vs. middle-onset group; *P*^b^: elderly-onset group vs. middle-onset group

The prevalence of comorbidities increased with age. Hypertension was present in 0% of early-onset, 10.4% of middle-onset, and 27.6% of elderly-onset patients. Diabetes mellitus was more frequent in the elderly-onset group compared to middle-onset groups (11.4% vs. 3.6%, *P* = 0.008). Depression/anxiety and anticholinergic medication use were low across all groups without significant differences.

Early-onset patients had a lower rate of abnormal Schirmer’s test (64.1%) than middle-onset patients (85.6%, *P* = 0.006), with no difference between middle- and elderly-onset groups (85.6% vs. 87.9%, *P* = 0.48).

Serological markers showed that anti-SSA positivity was similar across groups (66.8%-75.2%). Elderly-onset patients had higher anti-SSB (28.6% vs. 18.1%, *P* = 0.037) and anti-ANA positivity (69.5% vs. 53.9%, *P* = 0.009) than middle-onset patients. FS ≥ 1 was observed in > 90% of patients, with no group differences.

### Association between age and UWS flow rate

In univariate linear regression analysis, age was significantly associated with UWS flow rate. Each 10-year increase in age was associated with a decrease of 0.04mL/min in UWS flow rate (β= -0.04, 95% CI: -0.06 to -0.03, *P* < 0.001), indicating that UWS flow rate decreased with advancing age.

After adjusting for potential confounders including sex, pre-diagnostic period, and use of anticholinergic medications in multivariable linear regression, age remained independently associated with UWS flow rate (β= -0.04 per 10-year increase, 95% CI: -0.06 to -0.03, *P* < 0.001). In contrast, pre-diagnostic period (*P* = 0.45), sex (*P* = 0.24), and use of anticholinergic medications (*P* = 0.97) showed no significant independent associations with UWS flow rate.

### UWS flow rate according to age

Among patients with pSS, the median UWS flow rate was 0.20 mL/min (0.10–0.34), with 62.4% (211/338) falling below 0.25 mL/min. The early-onset group had a significantly higher UWS flow rate than the middle-onset group (0.29[0.20–0.47] vs. 0.20 [0.10–0.39] mL/min, *P* = 0.013), whereas the elderly-onset group had a significantly lower rate than the middle-onset group (0.14 [0.05–0.22] vs. 0.20 [0.10–0.39] mL/min, *P* < 0.001).

### Age-specific percentiles of UWS flow rate

To provide clinically useful reference values, percentiles of UWS flow rate were calculated for each age group (Table 2). In the early-onset group, the 5th, 25th, 50th, 75th, and 95th percentiles were 0.043, 0.20, 0.29, 0.47 and 0.99 mL/min, respectively. In the middle-onset group, the corresponding values were 0.037, 0.10, 0.20, 0.39 and 0.74 mL/min. In the elderly-onset group, the values were 0, 0.05, 0.14, 0.22 and 0.57 mL/min.

**Table 2:** Age-specific percentiles of UWS flow rate (mL/min)

Age group	*n*	5th	25th	50th	75th	95th
Early-onset group	40	0.043	0.20	0.29	0.47	0.99
Middle-onset group	193	0.037	0.10	0.20	0.39	0.74
Elderly-onset group	105	0	0.05	0.14	0.22	0.57

These data demonstrate a progressive downward shift in the distribution of UWS flow rate with increasing age. For example, the 50th percentile (median) in the elderly-onset group was approximately 52% lower than that in the early-onset group.

### Sensitivity analysis

The cohort included two pediatric patients under 18 years of age. A sensitivity analysis excluding these two patients was performed, and the results remained essentially unchanged: age remained significantly associated with UWS flow rate in multivariable regression (β= -0.04 per 10 years, *P* < 0.001), and median UWS flow rates progressively decreased with advancing age, consistent with the primary analysis-0.29 (0.20–0.46) mL/min in the early-onset group, 0.20 (0.10–0.39) mL/min in the middle-onset group, and 0.14 (0.05–0.22) mL/min in the elderly-onset group.

## Discussion

This study investigated the distribution of UWS flow rate across different age groups in patients with pSS and provided age-specific reference values for clinical settings. The main findings were: (1) UWS flow rate decreased significantly with advancing age, with each 10-year increase in age associated with a reduction of 0.04 mL/min; (2) elderly-onset patients had the lowest UWS flow rate, while early-onset patients had the highest; (3) the age-related decline in UWS flow rate was independent of sex, pre-diagnostic period, and anticholinergic medication use; and (4) the 5th, 25th, 50th, 75th, and 95th percentiles of UWS flow rate for each age group provide clinically useful reference values for longitudinal monitoring.

In earlier cohorts of patients with pSS-diagnosed according to the two most recent international classification criteria-the observed median UWS flow rate ranged from 0.08 to 0.21 mL/min [17, 19–21], consistent with our finding of 0.20 mL/min. This value falls below the range reported for adults in population-based studies (0.26–0.33 mL/min) [10]. Accordingly, in our cohort, 62.4% of patients had UWS flow rates below 0.25 mL/min-a value approximating the lower limit of the range reported by Bergdahl [10]-suggesting an adverse impact of pSS on salivary function and aligning with previous reports of impaired secretion in this population.

The clinical heterogeneity of pSS by age at onset has been increasingly recognized. Early-onset patients exhibit higher rates of hematological (80.56% vs. 52.53%, *P* = 0.001), renal (19.44% vs. 5.05%, *P* = 0.005), and mucocutaneous involvement (50% vs. 22.56%, *P* < 0.001), as well as a higher disease activity index (11 [6.25-17] vs. 7 [3–12], *P* = 0.003) despite shorter disease duration [5]. By contrast, elderly-onset pSS is characterized by a higher incidence of interstitial lung disease compared to both young-onset (48.1% vs. 14.3%, *P* < 0.001) [7] and typical middle-onset patients (7.9% vs. 5.7%, *P* = 0.035) [6]. Mirroring the age-related phenotypic divergence reported in the literature, our cohort also showed age-related differences in disease expression. Early-onset patients had shorter pre-diagnostic period and lower rate of abnormal Schirmer’s test than middle-onset group. Serologically, elderly-onset patients had higher anti-SSB and anti-ANA positivity than middle-onset group. These age-dependent clinical presentation features are not unique to pSS but parallel findings in other rheumatic diseases such as rheumatoid arthritis (RA) and systemic lupus erythematosus (SLE), where age at onset also influences disease severity and organ involvement [22, 23]. In RA, early-onset patients tend to have lower disease activity and less functional disability [22]; in SLE, early-onset is associated with more severe renal and neurological manifestations [23]. These shared patterns point to the possibility that age-related immune system changes may contribute to disease expression across autoimmune conditions.

We further examined whether UWS flow rate follows a similar age-dependent pattern. Our data showed that early-onset patients have higher UWS flow rate (0.29 mL/min), whereas elderly-onset patients exhibited the lowest rate (0.14 mL/min) in our cohort. This disparity is particularly evident when applying the conventional threshold of ≤ 0.1 mL/min: 85% of early-onset patients would be classified as having preserved salivary function, compared to only 55% of elderly-onset patients. However, given that early-onset patients demonstrate higher disease activity and more severe organ involvement (as detailed above), their relatively preserved UWS flow rate may mask true disease severity, potentially delaying timely intervention.

To address this issue, we established age-specific percentile reference values for UWS flow rate in a Chinese cohort with pSS-the first such study, to our knowledge. By providing age-appropriate reference points, our data may offer a practical tool for clinical judgments. For example, a 65-year-old patient with a UWS flow rate of 0.20 mL/min may be stable, while the same value in a 35-year-old patient may warrant concern. It is important to emphasize that these values are intended to complement, not modify, the 2016 ACR/EULAR classification criteria [14]. The ACR/EULAR criteria are widely recognized as a robust and validated framework that has greatly improved the standardization of pSS research and clinical trial enrollment, while also providing a reliable basis for identifying patients with pSS in clinical settings. Whereas these criteria serve an essential role in ensuring research consistency and diagnostic accuracy, our reference values aim to offer additional contextual information that may assist physicians in the day-to-day task of monitoring individual patients, without in any way diminishing the value or applicability of the established criteria.

An important consideration when interpreting age-related changes in salivary flow is the potential confounding effect of xerogenic medications, particularly anticholinergic agents. Commonly used for depression, overactive bladder, and other conditions, anticholinergic medications reduce salivary secretion by blocking muscarinic receptors in the salivary glands, thereby interrupting the neural stimulation of saliva production [24]. More than 600 medications possess anticholinergic properties [24], and a systematic review by Wolff et al. [25] identified 56 chemical substances with a higher level of evidence and 50 with a moderate level of evidence of causing salivary gland dysfunction or xerostomia, with anticholinergic agents representing one of the most commonly implicated drug classes. In older adults, a clear relationship has been demonstrated between anticholinergic drug use or anticholinergic burden and the presence of xerostomia [26]. In our cohort, 3.3% of patients were using anticholinergic medications at the time of saliva collection, with comparable rates across age groups. Importantly, the association between age and UWS flow rate remained significant after adjusting for anticholinergic use in multivariable analysis (β= -0.04 per 10 years, *P* < 0.001). Notably, this is not limited to anticholinergics. A recent large cohort study by the Xeromeds Consortium [16], which used both unmatched and matched analyses to control for age, sex, and concurrent medications, reported that no systemic medication had a statistically significant effect on UWS flow rate in 302 patients with SS, while age remained an independent predictor of reduced salivary flow (*P* = 0.005). Together, these findings suggest that anticholinergic medication use does not explain the observed age-related decline, and that systemic medications more broadly do not appear to be major confounders.

Disease duration is another important factor that may influence salivary flow in patients with pSS. Previous studies on disease duration and UWS flow rate have yielded conflicting results. Pijpe et al. [27] reported that salivary flow was higher in the early phase after diagnosis and decreased over time, suggesting a progressive decline with longer post-diagnostic disease duration. In contrast, Sluijpers et al. [28] found that once the diagnosis was established, UWS flow rate remained relatively stable during the disease course, with no significant further decline expected later in the disease. In the present study, we focused on the pre-diagnostic period, defined as the time from symptom onset to diagnosis. UWS flow rate was measured at the time of diagnosis. This definition captures the pre-diagnostic period but does not reflect post-diagnostic changes. After adjusting for pre-diagnostic period in multivariable regression analysis, age remained independently associated with UWS flow rate (β= -0.04 per 10 years, 95% CI: -0.06 to -0.03, *P* < 0.001), while pre-diagnostic period itself showed no significant independent association (*P* > 0.05). This finding suggests that over the pre-diagnostic period assessed in our cohort, the cumulative decline in UWS flow rate attributable to the length of this period is minimal, whereas age may play a more prominent role in determining salivary function. It is important to note, however, that this finding pertains only to the pre-diagnostic phase. This trajectory before diagnosis does not preclude the possibility of a more rapid decline immediately after diagnosis, as suggested by Pijpe et al. [27], nor does it contradict subsequent stabilization, as described by Sluijpers et al. [28] Collectively, these different trajectories may reflect distinct phases of disease progression.

Our cohort included 2 pediatric patients (< 18 years). Studies in pediatric SS have provided modest but informative data on salivary flow. In a multicenter cohort of 67 children with juvenile SS, Hammenfors et al. [29] reported mean UWS flow rates of approximately 0.37 mL/min in patients with normal salivary gland ultrasound findings, compared to 0.22 mL/min in those with pathologic findings (*P* = 0.049). Legger et al. [30] found a median UWS flow rate of 0.12 mL/min in children with pSS, compared with 0.07 mL/min in an adult cohort, though the difference was not statistically significant (*P* = 0.2). Given the small sample size in our study, we were unable to perform separate pediatric analyses; however, sensitivity analysis confirmed that exclusion of these patients did not alter the main findings. Nevertheless, our reference values should not be extrapolated to children.

This study has several limitations. First, the absence of a healthy control group and patients with other causes of reduced salivary flow limits interpretation of our results. Although we compared our findings with published normative data, such comparisons cannot fully account for potential differences in demographic characteristics, collection methods, or other unmeasured confounders between our cohort and the reference populations. Second, as a retrospective single-center study conducted at a Chinese tertiary hospital in an entirely Asian cohort, our findings-particularly the UWS reference values-may have restricted generalizability to other populations and settings, as salivary flow and pSS manifestations may vary with ethnicity, environment, and diet. Third, data on OSS were missing because this test was not routinely performed at our institution, which may have led to underdiagnosis of some pSS cases. Fourth, polypharmacy may have an additive effect on xerostomia/hyposalivation in elderly patients [31, 32]. As this study did not explore cumulative polypharmacy burden, we cannot fully exclude the possibility that polypharmacy may have partially contributed to the observed age-related decline in UWS flow rate, particularly in the elderly-onset group. If present, this bias would likely inflate the observed association between age and UWS flow rate. Fifth, the pre-diagnostic period was relatively short in our cohort, which may hinder correlation detection for the studied covariates and UWS flow rate. Finally, the number of patients with early-onset pSS included in this study was small, given that most cases of pSS occur after the age of 40, and the inclusion of only two pediatric patients was insufficient for a separate pediatric subgroup analysis.

Addressing these gaps, future studies should include healthy control groups and patients with other causes of reduced salivary flow to distinguish disease effects from physiological aging, and longitudinal designs with serial UWS assessments to map salivary flow trends over the disease course. Multicenter studies across diverse populations are needed to validate our reference values and explore ethnic variations. Larger cohorts of early-onset and pediatric patients are required to establish reference values for younger populations. Lastly, prospective management studies are required to evaluate whether implementing age-stratified UWS interpretation improves clinical outcomes.

## Conclusion

In conclusion, this study demonstrates that UWS flow rate in patients with pSS decreases significantly with advancing age, independent of sex, pre-diagnostic period, and anticholinergic medication use. These age-specific reference values may aid clinicians in interpreting UWS measurements based on patient age during follow-up. 

## Supplementary Information

Supplementary Material 1.

## Data Availability

All of the data supporting the conclusions of this article are included within the article.
